# Interplay Between Gut Microbiota and Cholesterol Metabolism in Colorectal Cancer

**DOI:** 10.3390/ijms27062553

**Published:** 2026-03-10

**Authors:** Sarah Wing Lam Li, Oscar Ting Hei Au, Effie Yin Tung Lau, Riley Yanjun Lu, Adrian Leonard Zaleski, Jessie Qiaoyi Liang

**Affiliations:** 1 Faculty of Medicine, The Chinese University of Hong Kong, Hong Kong, China; 1155175945@link.cuhk.edu.hk (S.W.L.L.); 1155193653@link.cuhk.edu.hk (O.T.H.A.); 2 Department of Medicine and Therapeutics, Li Ka Shing Institute of Health Sciences, CUHK Shenzhen Research Institute, The Chinese University of Hong Kong, Hong Kong, China; effielau@cuhk.edu.hk; 3 Schulich School of Medicine & Dentistry, Western University, London, ON N6A 3K7, Canada; ylu862@uwo.ca; 4 Department of Biology, University of Toronto, Mississauga, ON L5L 1C6, Canada; adrian.zaleski@mail.utoronto.ca

**Keywords:** colorectal cancer, gut microbiota, cholesterol metabolism, bile acid, oxysterol

## Abstract

Both gut microbiota dysbiosis and disrupted cholesterol metabolism are associated with colorectal cancer (CRC). While the interactions between these two factors have been well explored in diseases such as cardiovascular disease and atherosclerosis, their interactions and underlying mechanisms in CRC pathogenesis remain insufficiently explored, constituting a critical area for further investigation. This review examines the complex relationship between gut microbiota and cholesterol metabolism in CRC development from 2 perspectives: how specific gut microbial species can increase CRC risk by modulating cholesterol metabolism, particularly through bile acids and oxysterols, and how disrupted cholesterol metabolism can exacerbate microbial dysbiosis and promote CRC. The bidirectional relationship between gut dysbiosis and cholesterol dysregulation creates a vicious cycle that drives CRC development. Moreover, the potential of targeting the gut microbiome and cholesterol metabolism to develop new strategies for preventing and treating CRC is discussed, highlighting the promise of certain bacterial strains that exert protective effects via cholesterol-lowering mechanisms. By elucidating the intricate connections between gut microbiota, cholesterol metabolism, and CRC, this review paves the way for innovative approaches in CRC prevention and therapy.

## 1. Introduction

Colorectal cancer (CRC) is a major global health concern, ranking as the third most common cancer and the second leading cause of cancer-related deaths worldwide [1]. Its development is influenced by modifiable lifestyle factors, including obesity, physical inactivity, smoking, alcohol consumption, and poor dietary habits. Epidemiological studies suggest that dietary patterns significantly influence CRC risk, with diets rich in fiber, fruits, and vegetables exerting protective effects, whereas diets high in red/processed meats and fats increase risk [2]. High cholesterol has become a common health problem, raising the risks of various diseases, and has emerged as a key area of interest in CRC pathogenesis. While the direct impact of dietary cholesterol on CRC remains debated, accumulating evidence from prospective studies supports a positive association between elevated cholesterol levels and CRC development [3]. Proposed mechanisms include cholesterol-driven inflammation and oxidative stress, which are believed to contribute to CRC development. Another critical factor in CRC is the gut microbiota, a complex community of trillions of microorganisms with a vast collective genome called microbiome. Gut microbiota plays essential roles in intestinal homeostasis and immune regulation. Gut dysbiosis, an imbalance of the microbiota featuring increased pathogenic microbes and decreased beneficial microbes, has been strongly implicated in CRC, disrupting normal physiological processes and contributing to disease progression [4].

Recent research has highlighted a significant interplay between gut microbiota and cholesterol metabolism. Cholesterol metabolism, encompassing the synthesis, absorption, transport, and excretion of cholesterol and related metabolites, involves a complex interplay of various enzymes, receptors, and transport proteins. A healthy gut microbiota is essential for maintaining cholesterol homeostasis, whereas dysbiosis can alter bile acid (BA) metabolism and the conversion of cholesterol to coprostanol, which can potentially influence CRC risk [5]. Interestingly, disrupted cholesterol metabolism has also been reported to promote gut microbial dysbiosis associated with CRC [6]. These findings suggest that microbial composition and cholesterol metabolism engage in mutual regulation, collectively influencing CRC risk. However, the underlying mechanisms remain insufficiently explored.

This review aims to address existing knowledge gaps regarding how the interplay between gut microbiota and cholesterol metabolism influences CRC risk. We will summarize current research on: (1) how gut microbiota-mediated alterations in cholesterol metabolites, such as BAs and oxysterols, drive CRC pathogenesis; (2) how dysregulated cholesterol metabolism leads to gut dysbiosis associated with CRC; and (3) the potential of targeting the gut microbiota to improve cholesterol homeostasis in CRC management. By elucidating these relationships, we seek to advance the understanding of CRC mechanisms and identify novel strategies for preventing and treating CRC through modulation of gut microbiota-related cholesterol dysregulation.

## 2. Gut Dysbiosis Induces Dysregulated Cholesterol Metabolism to Promote CRC

### 2.1. Dysregulation of Cholesterol Biosynthesis by Gut Microbiota in CRC

Approximately 70% of cholesterol in the human body is derived from de novo synthesis, while only about 30% comes from dietary intake [7]. De novo cholesterol synthesis in human cells is tightly regulated by a negative feedback mechanism involving Sterol Regulatory Element-Binding Proteins (SREBPs). Among these, SREBP2 plays a critical role in regulating cholesterol synthesis in the colon and is implicated in colon cancer [8]. Additionally, transporter proteins that assist intestinal cells in absorbing dietary cholesterol are also regulated by SREBP2 [9].

Evidence indicates that specific CRC-associated pathogenic bacteria can promote CRC by modulating genes involved in cholesterol synthesis, particularly SREBP2. For instance, *Peptostreptococcus anaerobius* has been linked to CRC not only through the activation of pro-oncogenic pathways (e.g., AMPK, NF-κB, PI3K-Akt) but also specifically via its impact on cholesterol biosynthesis [10]. Mechanistically, *P. anaerobius* upregulates SREBP2 by inducing reactive oxygen species (ROS) via Toll-like receptor (TLR) 2 and 4, thereby promoting intracellular cholesterol synthesis. Pharmacological inhibition of SREBP2 can reverse *P. anaerobius*-induced cholesterol accumulation [11]. Collectively, *P. anaerobius* elevates intracellular cholesterol levels, fostering a proliferative microenvironment conducive to CRC initiation, progression, and metastasis.

Our recent study found that *Fusobacterium nucleatum* effectively promotes cholesterol synthesis in colon cells by inducing miR-130a-3p expression, which in turn suppresses AMPK activity by downregulating AMPKα/β1, leading to SREBP2 activation [12]. Furthermore, butyrate reduces cellular cholesterol levels by impairing SREBP2 signaling [13]. However, gut microbiota dysbiosis diminishes the abundance of butyrate-producing bacteria (e.g., *Faecalibacterium*, *Roseburia*), subsequently weakening this inhibitory effect on SREBP2.

Although whether other bacteria can similarly promote de novo cholesterol synthesis in human cells remains to be investigated, these findings underscore the role of gut dysbiosis in disrupting host de novo cholesterol biosynthesis and its connection to CRC.

### 2.2. De Novo Cholesterol Biosynthesis in Bacteria

Intriguingly, recent work suggests that certain bacteria can synthesize cholesterol de novo, challenging the traditional view of cholesterol as exclusively host- or diet-derived. A landmark study identified microbial sterol biosynthesis pathways in gut commensals, revealing bacterial cholesterol production as a previously unrecognized source [14]. Another study reported a significant change in the abundance of the cholesterol-producing bacterium *Enhygromyxa salina* in the fecal samples of patients with alcohol-induced osteonecrosis of the femoral head [15]. While the direct contribution of gut microbial-derived cholesterol to host dysmetabolism and CRC pathogenesis requires further investigation, these findings reveal an unappreciated complexity in bacterial sterol production and the source of cholesterol beyond de novo synthesis in the host and dietary intake. Bacterial sterol production could perturb local or systemic cholesterol homeostasis, potentially exacerbating dysbiosis-driven oncogenesis.

### 2.3. Gut Dysbiosis Dysregulates Cholesterol Esterification in CRC

Cholesterol esterification is a critical process for maintaining cellular cholesterol homeostasis and preventing lipotoxicity. This process is mediated by enzymes such as lecithin-cholesterol acyltransferase (LCAT) and sterol O-acyltransferase 1 (SOAT1, previously called ACAT1), which convert free cholesterol into cholesteryl esters for storage or incorporation into lipoproteins. In cancer cells with high cholesterol demand, cholesteryl esters can be hydrolyzed to replenish free cholesterol pools. Cholesteryl ester accumulation induced by PTEN loss and PI3K/AKT activation has been shown to contribute to cancer aggressiveness [16]. Notably, excessive SOAT1-mediated cholesterol esterification has been implicated in CRC progression [17]. Potential oncogenic roles of SOAT1 include immune evasion and TLR4 signaling crosstalk. SOAT1 inhibition enhances CD8+ T-cell proliferation and antitumor responses [18], suggesting that SOAT1 overexpression may suppress immune surveillance in CRC. These findings underscore the significance of cholesteryl ester and SOAT1 in CRC pathogenesis.

The loss of the tumor suppressor miR-148a upregulates ceramide synthase 5, leading to increased ceramide biosynthesis and disrupted gut microbial balance. This dysbiosis, in turn, triggers SOAT1 overexpression via TLR4 and β-catenin signaling, enhances cholesterol esterification, and ultimately promotes colorectal tumorigenesis in mice [19]. These findings position SOAT1 as a critical mediator linking gut dysbiosis, cholesterol esterification, and CRC progression. Clinically, high SOAT1 expression is strongly associated with lymph node metastasis and poor patient prognosis, resulting in unfavorable overall survival in CRC patients [17].

Furthermore, SREBP2, a key regulator of cholesterol synthesis, also plays a role in cholesterol esterification [20]. Therefore, gut dysbiosis-mediated activation of SREBP2 may additionally contribute to CRC pathogenesis through the dysregulation of cholesterol esterification.

### 2.4. Secondary Bile Acids Produced by Gut Bacteria Contribute to CRC

BAs are a group of 24-carbon acidic steroids that serve as the terminal products of cholesterol metabolism in hepatocytes. A portion of BAs (200–800 mg daily) escapes enterohepatic circulation and enters the colon lumen, where gut bacteria metabolize them into biologically active secondary forms [5].

The conversion of primary to secondary BAs involves three key enzymatic processes. First, bile salt hydrolases (BSHs) cleave the C-24 N-acyl amide bond, removing glycine or taurine moieties [21]. Metagenomic analyses indicate that BSHs are predominantly found in Firmicutes (30%), Bacteroidetes (14.4%), and Actinobacteria (8.9%) [22]. Notably, Gram-positive bacteria (e.g., *Clostridium*, *Bacteroidetes*, *Lactobacillus*, *Bifidobacterium*, *Enterococcus*, *Escherichia*, and *Listeria*) exhibit stronger BSH activity than Gram-negative bacteria, including certain archaea such as *Methanobrevibacter smithii* and *Methanosphera stadtmanae* [22]. Subsequently, hydroxysteroid dehydrogenases (HSDHs) mediate the oxidation and epimerization of 3-, 7-, and 12-hydroxy groups, with *Eggerthella lenta* being a documented contributor to these reactions [23]. Finally, 7α-dehydroxylation, carried out by specific gut microbes such as *Clostridium scindens* and *Eubacterium*, generates secondary BAs, including deoxycholic acid (DCA) and lithocholic acid (LCA) [24]. Additional bacterial species, such as *Desulfovibrionales*, *Ruminococcus*, and *Trichospiraceae*, also exhibit 7α-HSDH activity, with *Ruminococcus* abundance positively correlating with DCA levels [25].

Growing evidence suggests that these microbially derived secondary BAs play a significant role in the development and progression of CRC through multiple interconnected mechanisms. DCA and LCA induce oxidative stress and generate ROS, leading to DNA damage in colonic epithelial cells and contributing to carcinogenesis [26]. This view is supported by studies demonstrating that LCA induces single-strand breaks in the DNA of mouse colon crypt cells and inhibits polymerase β, which plays a role in DNA base excision repair [27]. Chronic exposure to elevated DCA levels promotes chromosomal instability and mutations in critical genes such as KRAS and TP53. This was demonstrated in an azoxymethane-treated rat model of colon tumorigenesis, where DCA increased the incidence of colon tumors and KRAS point mutations in those tumors. Additionally, frequent exposure of colonic epithelium to BAs favors cells resistant to BA-induced apoptosis, in which DCA achieves this by activating proteosomal degradation of the tumor suppressor p53 [27,28]. Moreover, secondary BAs, such as DCA, disrupt gut barrier integrity and promote chronic inflammation [29]. By increasing intestinal permeability, they facilitate bacterial translocation, triggering persistent inflammatory responses. These metabolites also stimulate the release of pro-inflammatory cytokines such as IL-6 and TNF-α, as well as COX-2, thereby creating a tumor-promoting microenvironment.

Recent studies have shifted focus from the idea of generalized inflammation as the primary driver of carcinogenesis to CRC-directed oncogenic signaling. DCA and LCA stimulate key signaling pathways, including EGFR, MAPK, and Wnt/β-catenin, enhancing cellular proliferation [30]. DCA promotes colon cancer cell proliferation and invasiveness by upregulating COX-2 through the epidermal growth factor receptor and activating the β-catenin cell-signaling pathway [28]. Furthermore, secondary BAs promote cancer stem cell development by modulating critical signaling pathways. For instance, DCA and LCA activate muscarinic 3 receptor (M3R) and Wnt/β-catenin signaling, resulting in a 12- to 15-fold upregulation of the oncoprotein c-Myc, which drives cancer stem cell transformation [31]. The oncogenic signaling pathways of CRC can also be driven by microbiota directly. The FadA adhesin from *F*. *nucleatum* regulates inflammatory and oncogenic genes by binding to E-cadherin, further activating the β-catenin signaling pathway [32].

### 2.5. Gut Microbiota Modulates Oxysterols to Influence CRC Risk

#### 2.5.1. Gut Microbiota-Mediated Oxysterol Production and Metabolism

Beyond cholesterol utilization, the body metabolizes cholesterol into various bioactive derivatives, including oxysterols. Oxysterols modulate cholesterol efflux through ATP-binding cassette (ABC) transporters, suppress LDL receptor expression, inhibit SREBP2-mediated cholesterol biosynthesis, and regulate cholesterol homeostasis via LXR activation. Furthermore, hepatic enzymes (e.g., CYP7A1) and gut microbiota can metabolize oxysterols into BAs, forming an interconnected metabolic network with profound implications for colorectal carcinogenesis [33,34].

Oxysterols arise via enzymatic oxidation and non-enzymatic oxidation pathways [35]. Gut microbiota is involved in generating oxidative stress, which in turn enhances oxysterol production through auto-oxidation of cholesterol in a non-enzymatic pathway. As a result, oxysterols have been a biomarker of oxidative stress [36]. Specific bacterial taxa upregulate NADPH oxidase 1 and Dual oxidase 2 in colonic enterocytes, elevating hydrogen peroxide levels [37]. Both commensal and pathogenic bacteria further amplify ROS through mitochondrial regulation, toxin production, or activation of formyl peptide receptors on macrophages [38]. This microbiota-driven oxidative environment promotes cholesterol auto-oxidation, particularly during dysbiosis, leading to the accumulation of pro-inflammatory, non-enzymatically derived oxysterols in the intestinal lumen.

#### 2.5.2. Cancer-Promoting Properties of Oxysterols

Given their dual roles as oxidative stress biomarkers and signaling mediators, oxysterols share oncogenic connections with BAs in CRC development [34]. Oxysterols contribute to colorectal carcinogenesis through three primary interconnected mechanisms, including inflammatory activation, epithelial barrier disruption, and tumor microenvironment modulation.

Oxysterols function as potent inflammatory mediators in the colon. Cholesterol oxidation products act as ligands for TLR2 and TLR4, triggering NF-κB-dependent release of pro-inflammatory cytokines, including TNF-α and IFN-β [39]. Notably, 25-hydroxycholesterol enhances IL-8 production by directly activating its promoter and synergistically amplifying IL-1β-induced IL-8 expression [40]. Oxysterol mixtures further stimulate a cascade of inflammatory mediators (IL-6, IL-23, MCP-1) and upregulate pattern recognition receptors (TLR2/9), fostering a chronic inflammatory milieu that promotes tumor growth and metastasis [41]. Additionally, 27-hydroxycholesterol suppresses anti-tumor immune responses by modulating T-cell activity [42].

On top of the pro-inflammatory effects, the pro-oxidant and cytotoxic properties of oxysterols lead to gut mucosal layer dysfunction [43]. Oxysterols also alter the colonic stroma by upregulating transforming growth factor-β (TGF-β) in fibroblasts and tumor-associated macrophages [44]. Furthermore, specific oxysterols exhibit direct oncogenic effects. 27-Hydroxycholesterol activates estrogen receptor beta (ERβ) and LXR, driving tumor growth, while suppressing anti-tumor immunity by modulating T-cell activity. It also upregulates MMP-9, enhancing invasion [45]. 5-Hydroxycholesterol potentiates Wnt/β-catenin signaling, a pivotal pathway in CRC progression [46]. 7-Ketocholesterol generates ROS, inducing DNA mutations and genomic instability [47]. 27-Hydroxycholesterol has been shown to act on immune myeloid cells residing at the distal metastatic sites, promoting an immune suppressive environment and facilitating breast cancer metastasis [48]. Collectively, the combined effects of chronic inflammation, epithelial barrier dysfunction, and stromal remodeling induced by oxysterols establish a permissive microenvironment for tumor development, thereby accelerating colorectal carcinogenesis.

#### 2.5.3. Anti-Cancer Properties of Oxysterols

Certain oxysterols have been reported to exhibit anti-cancer properties in specific contexts. For instance, in the colon cancer cell line Caco-2, specific oxysterols such as 7β-hydroxycholesterol and 5α,6α-epoxycholesterol induce apoptosis by promoting mitochondrial permeability transition and cytochrome c release [46,49], while 25-hydroxycholesterol triggers anoikis via activation of the p38-MAPK pathway [50]. However, due to limitations of these in vitro studies, the actual anti-cancer effect of these oxysterols in humans remains not fully answered.

### 2.6. Gut Dysbiosis Induces Dyslipidemia to Contribute to CRC

#### 2.6.1. Aberrant Cholesterol Transportation and Cellular Uptake in CRC

Cholesterol circulates in the bloodstream primarily as lipoprotein complexes, including low-density lipoprotein (LDL) and very-low-density lipoprotein (VLDL), which deliver cholesterol to peripheral tissues. Cellular uptake occurs via LDL receptor (LDLR)-mediated endocytosis, a process tightly regulated by cholesterol levels [51]. Under low intracellular cholesterol conditions, SREBP2 upregulates LDLR expression to enhance uptake. Conversely, high cholesterol levels suppress LDLR through LXR activation and proprotein convertase subtilisin/kexin type 9 (PCSK9)-mediated lysosomal degradation [52,53]. Notably, higher than normal LDLR expression is associated with cancer development, likely due to the heightened metabolic demands of proliferating tumor cells [54]. LDL and oxidized LDL (ox-LDL), generated via ROS-mediated LDL modification, further contribute to carcinogenesis through various mechanisms [55]. High-density lipoprotein (HDL), formed by apolipoprotein A-I binding with excess cholesterol, facilitates reverse cholesterol transport from peripheral tissues to the liver and steroidogenic sites for cholesterol elimination, metabolism or utilization. HDLs are typically reduced in CRC patients, though their precise role in carcinogenesis remains unclear [56].

#### 2.6.2. Aberrant Intracellular Trafficking and Efflux in CRC

Following cellular uptake, cholesterol is distributed via vesicular and non-vesicular transport mechanisms [7]. Of particular significance, abnormal cholesterol accumulation in mitochondria has been directly linked to neoplastic transformation [57]. To maintain cholesterol homeostasis, cells employ multiple efflux pathways mediated by ABC transporters. ABCA1 drives excess cholesterol to apolipoprotein A-I to form nascent HDL particles, while ABCG1 transports free cholesterol to mature HDL. ABCG5/8 promotes biliary cholesterol excretion and direct intestinal lumen secretion. It is reported to be significantly overexpressed in patients with advanced stages of CRC, playing a role in primary tumor growth and dissemination to distant sites [58]. Additionally, scavenger receptor class B type I (SR-BI) enables bidirectional cholesterol exchange via diffusion. Notably, dysregulation of ABCA1 and SR-BI has been implicated in CRC progression, suggesting disrupted cholesterol efflux as a potential oncogenic driver [58,59].

#### 2.6.3. Gut Dysbiosis and Dyslipidemia in CRC Pathogenesis

Dysbiosis-induced dyslipidemia arises from shifts in microbial composition, including reduced abundance of beneficial taxa (e.g., *Bifidobacterium*, *Lactobacillus*, and butyrate producers like *Fecalibacterium prausnitzii* and *Roseburia intestinalis*) and expansion of opportunistic pathogens (e.g., *Enterobacteriaceae*) [60]. High-fat diets exacerbate this imbalance, favoring endotoxin/lipopolysaccharide (LPS)-producing bacteria, leading to hyperlipidemia in rats [61,62]. Interventions such as red wine polyphenols can mitigate metabolic syndrome in obese patients by restoring beneficial bacteria, such as *Bifidobacteria*, *Lactobacillus* and butyrate-producing bacteria, and reducing LPS producers [63].

Gut dysbiosis influences lipid metabolism via the gut–brain axis, modulating appetite and satiety through immune and vagal signaling [64]. Dysbiosis disrupts this regulation, promoting metabolic dysfunction. Additionally, microbial imbalances trigger chronic low-grade inflammation via pro-inflammatory cytokines (e.g., TNF-α, IL-6, IL-1β) and adipokines (e.g., leptin), further exacerbating lipid dysregulation [65]. Increased intestinal permeability (“leaky gut”) permits bacterial endotoxin translocation, amplifying systemic inflammation and dyslipidemia [66].

Gut microbiota-derived metabolites, including BAs, LPS, and short-chain fatty acids (SCFAs), play pivotal roles in lipid homeostasis. BAs regulate lipid metabolism by activating TGR5 and farnesol X receptor (FXR), which promotes energy expenditure in adipose tissue and muscle and suppresses hepatic cholesterol synthesis respectively [66]. LPS enters circulation via a compromised gut barrier, inducing adipose tissue inflammation and lipid metabolism dysfunction [67]. Conversely, SCFAs modulate hepatic lipid enzymes, reduce serum triglycerides and cholesterol, and improve dyslipidemia through AMPK signaling and gut–brain axis communication [65,66]. SCFAs as a whole also acidify the lumen, speeding up excretion of bile by reducing solubility of BAs and hindering their conversion to secondary BAs.

**Figure 1:**
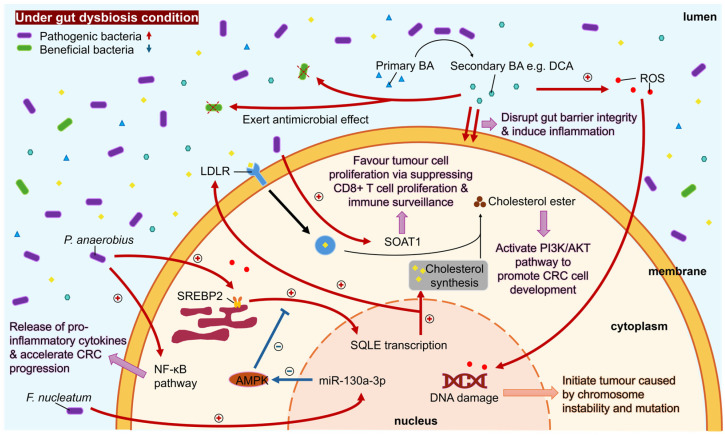
Summary of the effect of gut dysbiosis on cholesterol metabolism. Gut microbes exert effects at different stages of cholesterol synthesis, favoring cancer cell development.

Gut dysbiosis contributes to dyslipidemia, which in turn elevates CRC risk. A meta-analysis of prospective studies found that dyslipidemia—particularly elevated serum triglycerides and total cholesterol—was associated with increased CRC incidence, whereas higher HDL cholesterol exerted protective effects [68]. This is in view of the role dyslipidemia plays in intestinal inflammation, destruction of the protective mucous layer, and disruption of the balance between injury and recovery [68]. Thus, when dyslipidemia results from gut dysbiosis, CRC risk is inevitably raised. A summary of how gut dysbiosis affects cholesterol metabolism is illustrated in Figure 1.

## 3. Dysregulated Cholesterol Metabolism Leads to Gut Dysbiosis in Association with CRC

A pioneering study demonstrated that individuals with inherited hyperlipidemia exhibit gut dysbiosis, characterized by altered abundances of specific bacterial taxa, including *Lachnospiraceae*, *Ruminococcaceae*, *Akkermansia*, *Bacteroides*, *Roseburia*, and *Faecalibacterium* [69]. This finding suggests that dysregulated cholesterol levels can also modulate microbial composition. Such crosstalk between cholesterol metabolism and gut dysbiosis may ultimately contribute to CRC development.

### 3.1. Bile Acid-Induced Gut Dysbiosis in CRC

The role of BAs in inducing gut dysbiosis is linked to their antimicrobial properties. Free BAs have been shown to be antimicrobial, directly reducing intestinal bacterial populations in vivo [70]. DCA, the most typical secondary BA, exhibits the strongest antimicrobial activity, suppressing the growth of various intestinal bacteria, including *Clostridium perfringens*, *Bacteroides fragilis*, lactobacilli and bifidobacteria, highlighting its role in modulating gut microbiota composition [6]. DCA is derived from the bacterial 7α-dehydroxylation of the primary BA cholic acid. In wild-type rats, cholic acid administration increased *Firmicutes* (particularly *Clostridia*) while reducing *Bacteroidetes*, which was accompanied by impaired overall microbial diversity. This was noted alongside a proportional increase in DCA levels, further substantiating DCA’s role in modulating microbiota composition [71]. In *Apc^Min/+^* mice, DCA treatment altered gut microbiota composition and promoted intestinal carcinogenesis, an effect not observed in mice with antibiotic-mediated microbiota depletion [71].

Emerging evidence highlights how BA-induced dysbiosis exacerbates CRC risk by impairing intestinal barrier function and fostering low-grade inflammation. Free BAs disrupt membrane integrity, leading to the leakage of protons, potassium ions, and other cellular components, ultimately resulting in cell death [72]. The dysfunction of the intestinal barrier is intricately linked with colon tumorigenesis, as it worsens bacterial translocation, resulting in the production of pro-inflammatory cytokines and release of ROS. In *Apc^Min/+^* mice, DCA-induced gut dysbiosis was accompanied by impaired intestinal barrier, gut low-grade inflammation and tumor progression. Fecal microbiota transplantation from DCA-fed mice to *Apc^Min/+^* mice increased tumor multiplicity, induced inflammation, recruited tumor-associated macrophages and activated Wnt/β-catenin signaling pathway [6]. These findings support the role of BA-induced gut dysbiosis in CRC.

### 3.2. SQLE-Induced Gut Dysbiosis in CRC

SQLE, the rate-limiting enzyme in cholesterol biosynthesis, has been shown to promote carcinogenesis through direct stimulation of cell proliferation [73]. Furthermore, according to RNA sequencing analysis, SQLE was upregulated in tumor tissues of CRC patients and correlated with shorter survival [73]. Interestingly, SQLE also contributes to carcinogenesis through manipulation of the interplay between intestinal microbes and their metabolites, impairing normal gut barrier function [74]. Studies involving colon-specific SQLE transgenic mice and SQLE knockout mice have demonstrated that SQLE directly regulates gut microbiota composition by upregulating the cholesterol biosynthetic pathway, increasing intestinal cholesterol flux and secondary BA production. This shift favors a microbial environment rich in pathogenic bacteria and poor in beneficial microbes. Additionally, SQLE reduces tight junction proteins (Jam-c and occludin), affecting gut permeability. This impairment of gut barrier function induces epithelial inflammation, further contributing to gut dysbiosis [74].

### 3.3. Dysbiosis Caused by Oxysterols in CRC

Oxysterols similarly induce gut dysbiosis and impair intestinal barrier function. In C57BL/6J mice, 27-hydroxycholesterol has been shown to induce gut dysbiosis and intestinal barrier dysfunction by downregulating tight junction proteins (occludin, claudin-1/5, ZO-1), which widens intestinal junctions [75]. Dietary oxysterol mixtures can impair cognitive function through 27-hydroxycholesterol by causing microbiota dysbiosis and intestinal barrier dysfunction, highlighting the significant role of 27-hydroxycholesterol in modulating microbiota and contributing to disease development [76]. Furthermore, dietary oxysterol mixtures (including α-epoxy, β-epoxy, 7α-hydroxycholesterol, 7β-hydroxycholesterol and 7-ketocholesterol) activate TLR2/4 in enteric Caco-2 cells, disrupting tight junction proteins (occludin, claudin-1, JAM-A) [77]. Although oxysterol-induced dysbiosis has not yet been directly linked to CRC, a compromised intestinal barrier facilitates microbial translocation, perpetuating inflammation, dyslipidemia, and CRC progression.

## 4. Gut Microbiota Modulation to Improve Cholesterol Homeostasis

**Figure 2:**
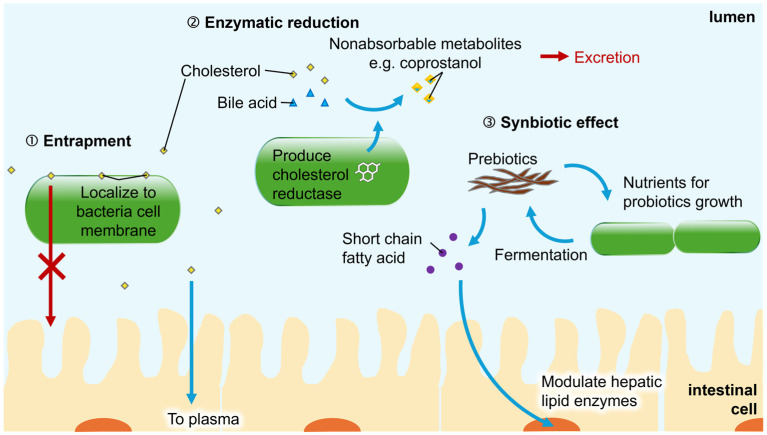
Cholesterol modulation via gut microbes. Cholesterol level is regulated by various mechanisms including entrapment, enzymatic reduction and synbiotics to achieve a balanced internal environment.

Given that gut dysbiosis contributes to CRC development through disrupted cholesterol metabolism, modulating the microbial community may play a key role in restoring cholesterol homeostasis and potentially reducing CRC risk. The following section summarizes the principal mechanisms by which gut bacteria regulate cholesterol levels (Figure 2).

### 4.1. Microbial Entrapment of Cholesterol

Certain gut bacteria can capture or sequester cholesterol and prevent its absorption or utilization by the host. This mechanism is believed to play a role in modulating cholesterol metabolism and reducing the availability of cholesterol for absorption into the bloodstream. Bacteria incorporate exogenous cholesterol into their cytoplasmic membranes in a similar way to that observed in sterol-non-requiring mycoplasmas [78]. Studies have been conducted to evaluate the efficacy of certain bacteria in reducing cholesterol levels. Lactobacilli and Bifidobacteria have been found to demonstrate cholesterol-assimilating activities, with cholesterol primarily localizing to their cellular membranes rather than being metabolically degraded [79,80]. This incorporation of cholesterol further alters the fatty acid composition of the bacterial membranes [81]. Notably, cholesterol assimilation is growth-dependent, as heat-killed bacteria exhibit reduced cholesterol-lowering capacity compared to viable cells [82]. Despite these findings, the clinical significance of microbial cholesterol entrapment in intestinal health and CRC prevention remains unclear. Further research is needed to elucidate its direct impact on CRC risk.

### 4.2. Microbial Enzymatic Reduction of Cholesterol

Besides direct cholesterol entrapment, gut bacteria utilize enzymes to break down cholesterol, potentially lowering its levels in the body. They convert cholesterol into nonabsorbable metabolites, such as coprostanol and coprostanone, which are excreted in feces. Microbial enzymatic reduction of cholesterol occurs via two main pathways. The first one involves a direct stereospecific reduction of the Δ5 double bond of cholesterol by gut bacteria. The second pathway is a three-step conversion involving the intermediates A4-cholestenone (II) and coprostanone (III) [83]. Although cholesterol-reducing bacteria were first identified in the 1930s, the specific microbial taxa responsible remain incompletely characterized. In humans, *Bacteroides* sp. strain D8 was the first isolated cholesterol-reducing bacterium [84], while *Eubacterium* spp. dominate in non-human models [85]. Subsequent studies have identified *Lactobacillus* and *Bifidobacterium* strains capable of partial cholesterol-to-coprostanol conversion [81,86]. Notably, high coprostanol levels in humans correlate with specific phylotypes from *Lachnospiraceae* and *Ruminococcaceae* [87]. Recent advances in metagenomics and biochemical assays have linked coprostanol production to a previously unrecognized clade of bacteria encoding the *IsmA* gene, a key enzyme in cholesterol metabolism [88]. This process further lowers blood cholesterol levels, as evidenced by an inverse correlation between fecal coprostanol/cholesterol ratios and serum cholesterol [89]. The aforementioned evidence consists of a mix of in vitro, animal and human observational studies, but the causal relationship between microbial enzymatic reduction of cholesterol and CRC is yet to be discovered.

### 4.3. Probiotics, Prebiotics, Synbiotics, and Postbiotics in Cholesterol Modulation

Certain probiotics have demonstrated cholesterol-lowering effects. Probiotics such as *Lactococcus* and *Lactobacillus* strains lower cholesterol by integrating it into their membranes during growth and converting it to coprostanol [81,90]. Additionally, *Lactobacillus* modulates host genes involved in cholesterol metabolism (e.g., NPC1L1, CYP7A1, ABCG5, ABCG8), thereby inhibiting intestinal cholesterol absorption [91]. Prebiotics, such as fructooligosaccharides (FOS), are non-digestible and fermentable ingredients that nourish beneficial bacteria and promote SCFA production, subsequently lowering serum lipid levels [92]. Soluble fibers (e.g., fructans) impede cholesterol absorption by forming viscous gels that physically block cholesterol uptake [93,94]. Synbiotics, which are combinations of probiotics and prebiotics, further enhance cholesterol-lowering effects. For instance, a synbiotic containing *Lactobacillus acidophilus* ATCC 4962, paired with inulin, FOS, and mannitol, has been shown to reduce plasma cholesterol levels [95]. Clinical studies indicate that synbiotic interventions can alter oxysterol profiles; for example, a combination of *Bifidobacterium longum* BB536 and red yeast rice extract reduces the oncogenic 27-hydroxycholesterol [96].

Recently, emerging studies have supported the notion that postbiotics possess the potential to reduce CRC risk through cholesterol modulation and their anti-tumor effects. Postbiotics refer to bioactive functional compounds produced during fermentation within a matrix that contribute to health benefits, including SCFAs, antioxidant enzymes, exopolysaccharides (EPS), bacterial cell wall fragments and lipoteichoic acid, bacterial lysates, and other metabolites [97]. EPS has been reported to exhibit cholesterol-lowering bioactivity and activate FXR-fibroblast growth factor 15 (FGF-15) axis [98,99]. SCFAs, such as butyrate and propionate, lower cholesterol by inhibiting de novo synthesis and exerting antitumor effects in CRC [13,100,101]. Furthermore, a study involving high-cholesterol rats fed postbiotic yogurt demonstrated a significant reduction in total cholesterol and LDL levels [102]. In addition to their anti-tumor effects in CRC, such as cell proliferation inhibition, apoptosis induction and cell cycle arrest, postbiotics reduce CRC risk by counteracting dyslipidemia and dysbiosis that are linked to CRC progression [103].

These findings (summarized in Table 1) underscore the potential of microbiota-targeted strategies, including probiotics, prebiotics, synbiotics, and postbiotics, to modulate cholesterol metabolism and mitigate oxysterol-driven CRC risk.

## 5. Discussion, Existing Hurdles, and Challenges

Integrating the cholesterol and microbial interactions into the classical adenoma–carcinoma progression model [106], the sequential mutations in APC, KRAS, and TP53 involved in different cholesterol-related pathways may lead to colorectal tumorigenesis at distinct stages. During the initiation stage, the mutational loss of APC triggers aberrant Wnt signaling, which fosters SREBP2 to increase de novo cholesterol synthesis and thus creates a proliferative microenvironment [107]. The resulting accumulation of secondary BAs and oxysterols also drives early DNA damage and chromosomal instability. As it progresses to the adenoma stage, driven by KRAS mutations, PCSK9 becomes critical by inhibiting LDLR degradation to enhance circulating cholesterol uptake for hyperproliferative demands [108]. At the stage of adenoma-carcinoma transition, invasion, and metastasis, driven by TP53 loss, SQLE and SOAT1 emerge as predominant cholesterol regulators. SQLE facilitates epithelial–mesenchymal transition by sustaining cholesterol flux for membrane remodeling, migration, and matrix invasion [109]. Meanwhile, SOAT1 converts free cholesterol into cholesteryl esters that are stored in lipid droplets, preventing lipotoxicity and enhancing the migration and invasion capabilities of CRC [17]. Gut dysbiosis and certain pathogens, such as *F*. *nucleatum*, linked to gene mutations, cancer pathway activations, and the upregulation of cholesterol-related genes, may also contribute to these interplays. However, elucidating the detailed profiles and mechanisms of specific features of gut dysbiosis involved in the various stages of adenoma–carcinoma progression warrants further investigation.

Besides the development of CRC from adenoma, different cholesterol mechanisms may contribute to distinct consensus molecular subtypes (CMSs) of tumors. CMS1 is associated with immune activation, while CMS2 exhibits high frequencies of WNT and MYC mutations. CMS3 is correlated with metabolic deregulation and KRAS mutations, while CMS4 features TGFβ activation [110]. Although few studies have explored the relationship between cholesterol and CRC subtypes, it has been indicated that a high proportion of cholesterol-rich patients, as well as CRC samples predominantly associated with cholesterol esterification, were classified as CMS4, suggesting a potential correlation with SOAT1 [111,112]. Certain pathways discussed earlier may share features with these subtypes, implying potential connections. WNT and MYC mutations in CMS2 may alter Wnt/β-catenin and PI3K/AKT signaling pathways [113,114]. Gut dysbiosis also influences these pathways, which may amplify the effects on cancer cell proliferation and CMS2 progression. The secondary BA DCA can promote KRAS mutations, which aligns with the characteristic of CMS3, assisting in the development of this tumor subtype. Distinct gut microbiome patterns have been associated with CMSs, such as *F*. *nucleatum* enrichment in CMS1 and CMS3 [115,116]. However, further investigation is needed to understand how gut dysbiosis interplays with dysregulated cholesterol metabolism to contribute to various CMSs. Moreover, it is worth conducting an in-depth investigation into whether targeting specific cholesterol pathways and the associated gut dysbiosis can aid in the management of different CMSs.

Further linking human cholesterol level to CRC risk, some case–control studies have highlighted a positive correlation of cholesterol level with CRC risk [117,118], while others did not find a significant association or even a negative relationship [119,120]. This paradoxical observation may be explained by different cancer stages with cachexic features, or altered metabolism and nutritional demands in patients, thereby causing fluctuating cholesterol levels among studies. Likewise, the effect of statins, medicines for lowering cholesterol levels, on CRC has been controversial. Several case–control studies and cohorts suggested a modest reduction in CRC risk by the use of statins, particularly in rectal cancer [121,122]. However, other studies also suggest long-term statin use is associated with increased risk of colon cancer [123,124]. This discrepancy can be attributed to factors such as the varied definitions of ‘‘long-term use’’ among the studies, or the types of statins used in different studies. Noticeably, lipophilic statins, e.g., simvastatin, may exert a more promising effect on CRC than hydrophilic statins, e.g., pravastatin, due to increased lipid solubility and membrane permeability [125]. Although the association of cholesterol/statin with CRC risk has been controversial with regard to the conflicting epidemiological studies, preclinical studies tend to be more conclusive in hypercholesterolemia’s promotion of CRC development, and it is widely accepted that cholesterol and CRC have a positive correlation.

One of the focuses of this review is the role of the gut microbiota in CRC progression through modulation of cholesterol metabolism. However, several limitations in current research should be noted. First, differences in BA profiles between animal models and humans complicate cross-species comparisons. Given the discrepancies in BA composition and feedback regulation between mice and humans, extrapolating findings from murine studies on the gut-microbiota−BA−host axis to human CRC pathogenesis requires caution. Second, most existing studies are observational in nature, emphasizing characterization of microbial alterations in CRC while overlooking causal relationships among gut microbiota, BA metabolism, and CRC development. Thus, further functional studies are needed to better elucidate host–pathogen interactions in colorectal carcinogenesis.

Some studies have advocated that BA signaling may have therapeutic benefits in suppressing molecular and phenotypic hallmarks of cancer progression [126,127,128]. However, these findings are based on non-CRC cell models. The BA−gut microbiome axis can influence cancer progression. It is possible that BAs function as potentially beneficial molecules in suppressing cancer progression if a physiological level of BA is attained, coupled with a healthy balanced approach to diet, medicine intake and other factors [129].

Although oxysterols have been shown to exhibit both cancer-promoting and anti-cancer effects, their net impact on CRC remains unclear based on current evidence. While in vitro studies indicate that individual oxysterols often demonstrate stronger anti-cancer effects compared to complex oxysterol mixtures [47,49,130], cytotoxic effects of oxysterols on different cell types vary by concentration and combinatorial action [40,49]. In the intestinal lumen, however, microbially driven autoxidation of dietary cholesterol under pathophysiological conditions generates intricate oxysterol mixtures. Consequently, the anti-tumor effects observed in controlled experimental settings may not fully translate to the human gut environment. Moreover, since cholesterol levels may alter significantly during cancer development, auto-oxidation and thus oxysterol concentration change accordingly. It can therefore be one of the hindrances in elucidating the positive or negative role of microbiota in CRC. Furthermore, some in vivo studies did not distinguish and identify different species of oxysterols mechanistically when presenting findings [131]. As these oxysterols can originate from diet or enzymatic modulation in addition to microbiota, it is difficult to tell exactly the net contribution of microbiota to oxysterols and thus CRC and hence utilize the knowledge in diagnosis, prevention, or therapy. There are perhaps some gaps to be filled, including the individual fluctuation of oxysterol concentration over time, variations among individuals and the exact contribution of microbiota within the spectrum of oxysterols that our cells respond to.

Finally, we touched upon the role of the microbiota in mitigating CRC risk by improving cholesterol homeostasis. Despite this being a long-standing belief proposed more than 100 years ago, few studies have delved into this relation. In reality, the gut microbiota involved in the conversion of cholesterol to coprostanol or its other saturated analogs is diverse and encompasses various bacterial species. A large-scale multi-omics study demonstrated that microbes encoding 3β-HSDH, an enzyme in the first step of cholesterol reduction to coprostanol, are frequent and abundant in geographically diverse human microbiomes [88]. However, efforts to fully grasp the complete picture are hindered in light of the difficulty in culturing certain microbial species that are involved in this metabolic process and the limited biochemical and genetic knowledge regarding this metabolic process. To date, the exact organisms that give rise to the conversion of cholesterol to coprostanol have still not been comprehensively determined.

## 6. Conclusions

Our review synthesizes current evidence on how gut microbiota and cholesterol metabolism interact collectively to influence CRC. Gut microbiota gives rise to CRC via dysregulation of cholesterol metabolism including its biosynthesis, export, esterification, and trafficking. Key microbial species that contribute to CRC through modulation of cholesterol metabolism are summarized in Table 2. In particular, *P. anaerobius* and *F. nucleatum* have strong associations with CRC through cholesterol overproduction. Notably, while alteration of bacterial composition leads to aberrant cholesterol homeostasis and dyslipidemia, cholesterol dysregulation can further aggravate gut dysbiosis. This may result in a vicious cycle, further promoting cancer development and progression.

We have also discussed the potential of targeting the gut microbiota to modulate cholesterol metabolism as a strategy for reducing CRC risk. Due to current research limitations, this review has primarily focused on the roles and mechanisms of specific bacterial species, key microbial enzymes, and probiotics in cholesterol regulation. Nonetheless, other approaches aimed at correcting cholesterol metabolic disorders and gut microbiota dysbiosis may also represent promising strategies for CRC prevention. BA sequestrants, such as cholestyramine, can bind secondary BAs, thereby reducing their carcinogenic effects. A Western-style diet (high in fat) promotes the secretion of primary BAs, which are then converted by gut microbiota into carcinogenic secondary BAs, further increasing CRC risk. Low-fiber diets impair the binding capacity for secondary BAs, leading to elevated levels of free secondary BAs in the colon, whereas high-fiber diets facilitate BA binding and reduce their toxicity. Therefore, adopting a healthy diet—reducing fat intake and increasing dietary fiber—can suppress secondary BA production and help shape a healthier gut microbiome, ultimately lowering CRC risk. Additionally, fecal microbiota transplantation may help restore a balanced gut microbiota and modulate cholesterol metabolism, contributing to CRC prevention.

There are several critical questions that warrant further investigation given the promising but insufficient knowledge demonstrated by current studies. First, little is known about the precise mechanisms by which specific bacterial strains may impact CRC risk by raising or lowering cholesterol levels. Future studies should focus on identifying which bacteria can influence host cholesterol metabolism and elucidating their underlying pathways. Second, as evidence accumulates regarding BAs affecting CRC risk via the BA-microbiota axis, an emerging research direction involves developing strategies to selectively target and eliminate excess BAs without disrupting normal signaling functions. Finally, in addition to addressing the aforementioned knowledge gaps concerning oxysterols, it is essential to investigate whether cholesterol-lowering bacterial strains or probiotics can selectively reduce pro-carcinogenic oxysterols in the gut and evaluate their net impact on CRC. Future research should further elucidate the connection between microbiota, oxysterol and cholesterol alleviation to facilitate the development of more effective strategies for CRC prevention.

## Figures and Tables

**Table 1:** Summary of the effects of probiotics, prebiotics, synbiotics, and postbiotics on cholesterol modulation.

Categories	Components	Cholesterol Effects/Mechanism	References
Probiotics	*Lactobacillus*	↓ Cholesterol	[81,91]
	*Lactococcus lactis*	↓ Total Cholesterol, ↓ Triglyceride, ↑ HDL	[90]
Prebiotics	FOS	Promotes SCFAs → ↓ cholesterol	[92]
	Fructans	↓ Intestinal uptake	[93,94]
Synbiotics	*Lactobacillus acidophilus* + inulin/FOS/mannitol	↓ Cholesterol	[95]
	*Bifidobacterium longum* + red yeast rice	↓ Oncogenic 27-hydroxycholesterol	[96]
	*Lactobacillus pentosus* + *Enterococcus faecalis* + lactulose	↓ Cholesterol	[104]
	*Lactobacillus salivarius* + FOS	↓ TC & LDL	[105]
Postbiotics	EPS-D1	↓ Total Cholesterol, triglyceride & LDL	[99]
	Butyrate	Inhibit cholesterol biosynthesis	[100]
	Propionate	↓ Cholesterol	[101]

**Table 2:** Key microbial species whose dysregulation contributes to CRC through the modulation of cholesterol.

Microbial Species	Metabolic Mechanism	Pathogenic Pathway	Effect on CRC
*Peptostreptococcus anaerobius*	Induces intracellular de novo cholesterol biosynthesis	Interacts with TLR2/TLR4 to induce ROS, which upregulates SREBP2	Fosters a proliferative microenvironment conducive to CRC initiation, progression, and metastasis
*Fusobacterium nucleatum*	Enhances cholesterol biosynthesis	Induces miR-130a-3p expression, suppressing AMPK activity and leading to SREBP2 activation	Promotes tumor cell proliferation
*Bacteroides*, *Clostridium*, *Listeriaceae*	Deconjugates primary BAs via strong BSH activity	Increases the pool of unconjugated BAs, providing substrates for 7α-dehydroxylation	Facilitates the production of pro-carcinogenic secondary BAs; promotes intestinal inflammation
*Clostridium scindens*, *Eubacterium* spp.	Converts primary BAs to secondary BAs (DCA, LCA)	Utilizes the 7α-dehydroxylation pathway to produce DCA and LCA	Induces DNA damage, oxidative stress, chromosomal instability, mutations in critical genes like KRAS, and cellular proliferation
*Lactobacillus* spp., *Bifidobacterium* spp.	Lowers serum cholesterol and LDL levels	Converts cholesterol into the non-absorbable coprostanol; reduces intestinal cholesterol absorption	Mitigates CRC risk by reducing the “fuel” for tumor cell membranes and oncogenic signaling

## Data Availability

No new data were created or analyzed in this study. Data sharing is not applicable to this article.

## Abbreviations

The following abbreviations are used in this manuscript:

ABC	ATP-binding cassette
BA	Bile acid
BSH	Bile salt hydrolase
CMS	Consensus molecular subtype
CRC	Colorectal cancer
DCA	Deoxycholic acid
ERβ	Estrogen receptor beta
EPS	Exopolysaccharides
FOS	Fructooligosaccharides
HDL	High-density lipoprotein
HSDH	Hydroxysteroid dehydrogenase
LCA	Lithocholic acid
LCAT	Lecithin-cholesterol acyltransferase
LDL	Low-density lipoprotein
LDLR	Low-density lipoprotein receptor
LPS	Lipopolysaccharide
M3R	Muscarinic 3 receptor
ox-LDL	Oxidized low-density lipoprotein
PCSK9	Proprotein convertase subtilisin/kexin type 9
ROS	Reactive oxygen species
SCFA	Short-chain fatty acid
SOAT1	Sterol O-acyltransferase 1
SR-BI	Scavenger receptor class B type I
SREBP	Sterol Regulatory Element-Binding Protein
TGF-β	Transforming growth factor-β
TLR	Toll-like receptor
VLDL	Very-low-density lipoprotein
